# Metabolomic profiling reveals novel biomarkers and therapeutic targets in Legg-Calvé-Perthes disease: a comprehensive analysis of peripheral blood and endothelial function

**DOI:** 10.3389/fphys.2025.1641445

**Published:** 2025-10-01

**Authors:** Shaoneng Zi, Chengyong Wang, Tong Zhang, Qian Lv, Zhiying Wan, Pengju He, Yong Hang, Yongqing Xu

**Affiliations:** ^1^ Department of Orthopedics, The 920th Hospital of Chinese People’s Liberation Army Joint Logistics Support Force, Kunming, Yunnan, China; ^2^ Department of Orthopedics, Kunming Children’s Hospital, Kunming, Yunnan, China

**Keywords:** Legg-Calvé-Perthes disease, metabolomics, vascular dysfunction, inflammation, endothelial cells, 3-ketoglucose, sanguinarine

## Abstract

**Introduction:**

Legg-Calvé-Perthes disease (LCPD) is juvenile idiopathic femoral head avascular necrosis with unclear pathophysiology. We aimed to identify circulating metabolic biomarkers and clarify the roles of peripheral inflammation and vascular/endothelial dysfunction in LCPD, and to evaluate the protective potential of 3-ketoglucose (3-KG) and sanguinarine (SANG).

**Methods:**

Peripheral blood from children with LCPD (n=36) and healthy controls (n=6) underwent untargeted LC-MS metabolomics with differential and pathway analyses. Candidate metabolites (3-KG, SANG) were tested in LPS-challenged HUVECs for effects on viability, ROS, IL-1β/IL-6/TNF-α, and NF-κB/eNOS/VCAM-1 (RNA-seq, qPCR, Western blot, immunofluorescence). *In vivo* validation used a steroid/LPS-induced rat model of femoral head osteonecrosis assessing histology, adipogenesis, serum ALP/TG, and Nos3/Vcam1/Nfkb1 expression.

**Results:**

Thirty-eight metabolites differed significantly between LCPD and controls; 3-KG and SANG were upregulated, whereas several metabolites including N-methyl-D-aspartate were downregulated, mapping to inflammatory and oxidative-stress pathways. Both 3-KG and SANG dose-dependently mitigated LPS-induced HUVEC injury by restoring viability, lowering ROS and pro-inflammatory cytokines, and normalizing NF-κB/eNOS/VCAM-1 at mRNA and protein levels, with SANG showing greater potency. In rats, both compounds ameliorated bone loss and adipogenesis, increased ALP, reduced TG, and reversed MPS-induced changes in Nos3, Vcam1 and Nfkb1.

**Discussion:**

This work defines a peripheral “metabolomic fingerprint” of LCPD and links systemic metabolic alterations to endothelial inflammation/dysfunction. 3-KG and SANG exhibit endothelial-protective activity *in vitro* and *in vivo*, supporting their promise as diagnostic biomarkers and therapeutic candidates. Larger, longitudinal cohorts are needed to validate these signatures and clarify stage-specific dynamics.

## 1 Introduction

Legg-Calvé-Perthes Disease (LCPD) is an idiopathic osteonecrosis of the juvenile femoral head, presenting a significant orthopedic challenge in children typically aged 3–12 years (Laine et al., 2021; Rodríguez-Olivas et al., 2022). The interruption of blood supply to the proximal femoral epiphysis leads to bone death, potential structural collapse, and subsequent deformity, which can result in long-term complications such as premature osteoarthritis and impaired hip function. Despite extensive research, the precise etiology and complete pathophysiological cascade of LCPD remain incompletely understood, underscoring the need for more profound molecular insights (Maleki et al., 2021). The disease classically progresses through necrosis, fragmentation, reossification, and remodeling, processes that are influenced by a complex interplay of biological factors (Ng et al., 2024).

A critical element in LCPD pathogenesis is profound vascular dysfunction, leading to ischemia of the femoral head. Growing evidence indicates that endothelial dysfunction is a central component of vascular injury in LCPD; for example, increased levels of circulating endothelial microparticles (EMPs), which directly inhibit endothelial cell function, have been detected in patients with Perthes disease (Li et al., 2021). Furthermore, there is growing evidence that peripheral inflammation is modulated in LCPD, potentially exacerbating vascular injury and affecting bone healing processes. The interplay between inflammatory mediators, endothelial cell activation (as often studied using models like Human Umbilical Vein Endothelial Cells (HUVECs)) (Elomaa et al., 2022; Yang et al., 2023), and local tissue responses is an area of active investigation.

Current diagnostic approaches for LCPD primarily rely on imaging, and treatment strategies aim to maintain femoral head sphericity and prevent long-term disability (Ng et al., 2024), yet a clear consensus on optimal management is lacking, partly due to an incomplete understanding of the disease at a molecular level (Leroux et al., 2018). The identification of reliable biomarkers for early diagnosis, prognostic stratification, or monitoring treatment response is a significant unmet need (Zhang et al., 2023). High-throughput ‘omics’ technologies, such as metabolomics, offer a promising avenue to explore the molecular underpinnings of complex diseases (Jang et al., 2018). Metabolomic profiling can provide a functional readout of cellular and systemic physiological states, with studies demonstrating its potential to reveal alterations in critical pathways related to energy metabolism, oxidative stress, and inflammation in various conditions, including those affecting musculoskeletal health (de Moraes et al., 2023). However, comprehensive metabolomic investigations in LCPD, particularly those linking systemic metabolic signatures to local pathophysiological events like endothelial dysfunction, have been limited (Rodríguez-Olivas et al., 2022).

This study, therefore, was designed to investigate the metabolic landscape of LCPD through a comprehensive analysis of peripheral blood from patients and healthy controls. We aimed to identify differential metabolites that could serve as novel biomarkers and to elucidate their potential roles in the context of endothelial inflammation and vascular dysfunction, key contributors to LCPD pathology. By exploring these connections, we hope to uncover new therapeutic targets and enhance the understanding of this enigmatic pediatric disease.

## 2 Materials and methods

### 2.1 Study design and patient recruitment

This prospective, clinical observational study strictly adhered to the Declaration of Helsinki and relevant ethical guidelines. All study protocols were formally approved by the Ethics Committee of the 920th Hospital of the Joint Logistics Support Force of the Chinese People’s Liberation Army (Approval No. Ethics Review 2015–017(Section)-01). Before conducting any study procedures, a detailed explanation of the study was provided to the parents or legal guardians of all child participants, and their written informed consent was obtained. This ensured they fully understood the study’s purpose, procedures, potential risks, and benefits.

Patients were recruited from the outpatient and inpatient departments of the 920th Hospital of the Joint Logistics Support Force of the Chinese People’s Liberation Army. Following sample collection, metabolomic analyses and endothelial cell function validations were performed, with data analysis subsequently completed.

Inclusion criteria, as per the study protocol, were: (1) age 4–12 years; (2) unilateral Legg-Calvé-Perthes Disease (LCPD) confirmed by X-ray and magnetic resonance imaging (MRI), with disease duration and<6 months from initial diagnosis; (3) no prior LCPD-related surgical or pharmacological treatment; (4) absence of severe systemic diseases or infections, and a mental state allowing cooperation; (5) signed informed consent.

Exclusion criteria comprised: (1) history of other skeletal, metabolic, or hereditary diseases; (2) previous LCPD treatment; (3) presence of severe systemic diseases (e.g., cardiac, hepatic/renal insufficiency, immunodeficiency), other inflammatory diseases, or acute infections; (4) concurrent use of medications potentially interfering with study outcomes (e.g., immunosuppressants, corticosteroids, anti-inflammatory drugs); (5) inability to provide informed consent or meet other ethical trial requirements.

Thirty-six LCPD patients meeting these criteria and six age- and sex-matched healthy control children (recruited during routine health examinations) were enrolled. Standardized case report forms were used for data collection.

### 2.2 Sample collection and processing

Peripheral blood (5 mL) was collected via venipuncture into EDTA tubes between 8:00–10:00 a.m. after an overnight fast. Samples were processed within 2 h: plasma was separated by centrifugation (3,000 rpm, 15 min, 4 °C), aliquoted, and stored at −80 °C until analysis.

### 2.3 Metabolomic analysis

Plasma samples (50 mg) were extracted with 400 μL methanol: water (4:1, v/v) containing L-2-Chlorophenylalanine (internal standard). The mixture was ultrasonicated (40 kHz, 5 °C, 30 min), precipitated at −20 °C for 30 min, and centrifuged (13,000 × g, 15 min, 4 °C). The supernatant was analyzed by LC-MS/MS.

Separation utilized a Vanquish UHPLC and Orbitrap Exploris 120 mass spectrometer (Thermo Fisher Scientific) with an ACQUITY UPLC® HSS T3 column (1.8 µm, 2.1 × 100 mm; Waters) at 40 °C. Mobile phase A was 0.1% formic acid in water, and B was 0.1% formic acid in acetonitrile (flow rate: 0.3 mL/min). The gradient was: 0–5 min, 10%–98% B; 5–6.5 min, 98% B, then re-equilibration.

### 2.4 *In Vitro* experiments

#### 2.4.1 Cell culture

Human Umbilical Vein Endothelial Cells (ATCC® PCS-100–010^TM^) were cultured in EGM-2 medium (Lonza) with 10% FBS and 1% penicillin-streptomycin at 37 °C, 5% CO_2_. Passages 3–6 were used.

Cells were seeded (2 × 10^5^/well in 6-well plates for protein/RNA; 5 × 10^4^/well in 24-well plates for immunofluorescence) and adhered for 24 h. To systematically evaluate the dose-dependent effects and inherent cytotoxicity of the compounds, HUVECs were assigned to the following treatment groups: (1) Control (vehicle only); (2) LPS (100 ng/mL; Sigma-Aldrich); (3) 3-ketoglucose (3-KG) alone (10, 20, 40 μg/mL); (4) Sanguinarine (SANG) alone (10, 20, 40 μg/mL); (5) Pre-treatment with 3-KG (10, 20, or 40 μg/mL for 2 h) followed by LPS stimulation; (6) Pre-treatment with SANG (10, 20, or 40 μg/mL for 2 h) followed by LPS stimulation. The concentrations were selected based on preliminary screening to establish a non-toxic working range and to assess dose-response relationships.

#### 2.4.2 Cell viability and proliferation assays

Viability was assessed using CCK-8 (Dojindo). HUVECs (5 × 10^3^/well, 96-well plates) were treated, then incubated with CCK-8 solution (10 μL/well, 2h, 37 °C). Absorbance (450 nm) was read. Proliferation was evaluated using an EdU incorporation assay (Beyotime) per manufacturer’s instructions.

#### 2.4.3 ROS and inflammatory cytokine detection

Intracellular ROS was measured using DCFH-DA (10 μM; Beyotime). HUVECs were incubated (30 min, 37 °C, dark). Fluorescence (Ex: 485 nm; Em: 535 nm) was quantified and imaged.

Supernatant IL-1β, IL-6, and TNF-α levels were quantified using ELISA kits (R&D Systems; Cat. Nos. DLB50, D6050, DTA00D) per manufacturer’s instructions. Optical density was read at 450 nm (correction at 570 nm).

### 2.5 RNA extraction and transcriptome sequencing

Total RNA was extracted using TRIzol (Invitrogen). RNA quality (RIN >8.0, OD260/280 1.8–2.0) was confirmed (Agilent 2,100 Bioanalyzer; NanoDrop 2000). Library preparation used NEBNext® Ultra^TM^ II RNA Library Prep Kit (NEB). Briefly, mRNA was enriched, fragmented, and reverse transcribed. Second-strand synthesis was followed by end repair, A-tailing, adapter ligation, and PCR. Paired-end 150 bp reads were generated on an Illumina NovaSeq 6,000. Raw reads were quality-controlled (FastQC), filtered, and clean reads aligned to GRCh38 (STAR aligner). Gene expression was quantified (featureCounts) and normalized to FPKM.

### 2.6 Immunofluorescence analysis

HUVECs on coverslips (70% confluence) were treated, fixed (4% paraformaldehyde, 15 min), and permeabilized (0.1% Triton X-100, 10 min). After blocking (5% BSA, 1 h), cells were incubated with primary antibodies (*NF-κB, eNOS, VCAM-1*; 1:200 each; Abcam) overnight at 4 °C, then with fluorescence-conjugated secondary antibodies (1:500; Invitrogen) for 1 h at RT. Nuclei were DAPI-stained (Beyotime). Images were captured (Olympus FV3000 confocal microscope) and analyzed (ImageJ).

### 2.7 Western blot analysis

Total protein was extracted (RIPA buffer; Beyotime, with inhibitors; Roche) and quantified (BCA assay; Thermo Fisher Scientific). Protein (30 μg) was separated by SDS-PAGE and transferred to PVDF membranes (Millipore). Membranes were blocked (5% non-fat milk/TBST, 1 h), then incubated with primary antibodies (anti-*NF-κB p65*, *anti-eNOS*, *anti-VCAM-1*, 1:1000 each; anti-GAPDH, 1:5000; Cell Signaling Technology) overnight at 4 °C. After HRP-conjugated secondary antibody incubation (Beyotime), bands were visualized (ECL; Millipore) and quantified.

### 2.8 Quantitative real-time PCR

cDNA was synthesized (1 μg RNA; PrimeScrip^TM^ RT Reagent Kit; Takara Bio). qPCR used TB Green® Premix Ex Taq^TM^ (Takara Bio) on a LightCycler 480 II (Roche). Conditions: 95 °C for 30s; 40 cycles of 95 °C for 5s, 60 °C for 30s. Relative gene expression was normalized to GAPDH using the 2^-ΔΔCt^ method. Primers are in Supplementary Tables S1,S2.

### 2.9 Animal experiments

#### 2.9.1 Animal model and grouping

A total of forty healthy 8-week-old female Sprague-Dawley (SD) rats, weighing approximately 220–250 g, were purchased from the Experimental Animal Center of Kunming Medical University. All animal procedures were approved by the Institutional Animal Care and Use Committee (IACUC). After 1 week of acclimatization, the rats were randomly divided into four groups (n = 10 per group): a Control group, an MPS model group, an MPS + 3-ketoglucose treatment group, and an MPS + Sanguinarine treatment group.

To establish the osteonecrosis of the femoral head (ONFH) model, rats in the model and treatment groups first received an intravenous injection of lipopolysaccharide (LPS, Sigma) via the tail vein at a dose of 10 μg/kg/d for two consecutive days. Twenty-four hours after the first LPS injection, they were administered an intramuscular injection of methylprednisolone (MPS) at a dose of 20 mg/kg/d for three consecutive days.

Based on our preliminary laboratory data, rats in the treatment groups received daily intraperitoneal (i.p.) injections starting from the same day as the first MPS injection. The MPS + 3-ketoglucose group was administered 3-ketoglucose at 30 mg/kg, and the MPS + Sanguinarine group was administered sanguinarine at 20 mg/kg. The MPS group received daily i. p. Injections of the vehicle, while the Control group received equivalent volumes of normal saline injections at the corresponding time points. All interventions were continued for 12 weeks. At the end of the treatment period, blood samples were collected from the tail vein for biochemical analysis. Rats were then euthanized, and femoral head samples were harvested.

#### 2.9.2 Serum ALP and TG

Blood samples were centrifuged at 5,000 rpm for 10 min at 4 °C. The serum was separated, and serum alkaline phosphatase (ALP) activity was measured using a specific assay kit (Nanjing Jiancheng Bioengineering Institute, China) according to the manufacturer’s instructions. The absorbance at 405 nm was recorded to determine the relative ALP levels. Serum triglyceride (TG) levels were measured using a fully automated biochemical analyzer (Roche Modular-T; Roche, Basel, Switzerland).

#### 2.9.3 Histological and immunohistochemical analyses

The harvested femoral heads were decalcified and embedded in paraffin. They were then sectioned in the coronal plane at a thickness of 5 μm. Sections from the left femoral head of each group were stained with hematoxylin and eosin (H&E). Oil Red O staining was used to detect lipid droplet formation during adipogenesis. After fixation and pre-wetting with 60% isopropanol, femoral head sections were stained with Oil Red O working solution for 15 min. The positive areas were quantified using ImageJ 1.47 software. The diagnosis of osteonecrosis was based on the presence of empty lacunae or pyknotic nuclei of osteocytes in the bone trabeculae, accompanied by surrounding bone marrow necrosis.

### 2.10 Statistical analysis

Data from ≥3 independent experiments are mean ± SD. Analyses used GraphPad Prism 8.0.2. Normality was assessed (Shapiro-Wilk test). Two-group comparisons used Student’s t-test or the Mann-Whitney U test. Multiple groups were compared using one-way ANOVA (Tukey’s *post hoc*) or Kruskal-Wallis test (Dunn’s *post hoc*). Correlations used Pearson’s or Spearman’s coefficients. P < 0.05 denoted significance. Power analysis (G*Power 3.1; effect size = 0.8, α = 0.05, power = 0.9) confirmed adequate sample size. Analyses were blinded.

## 3 Results

### 3.1 Global metabolomic profiling and multivariate statistical analysis reveal distinct metabolic signatures in LCPD

**FIGURE 1:**
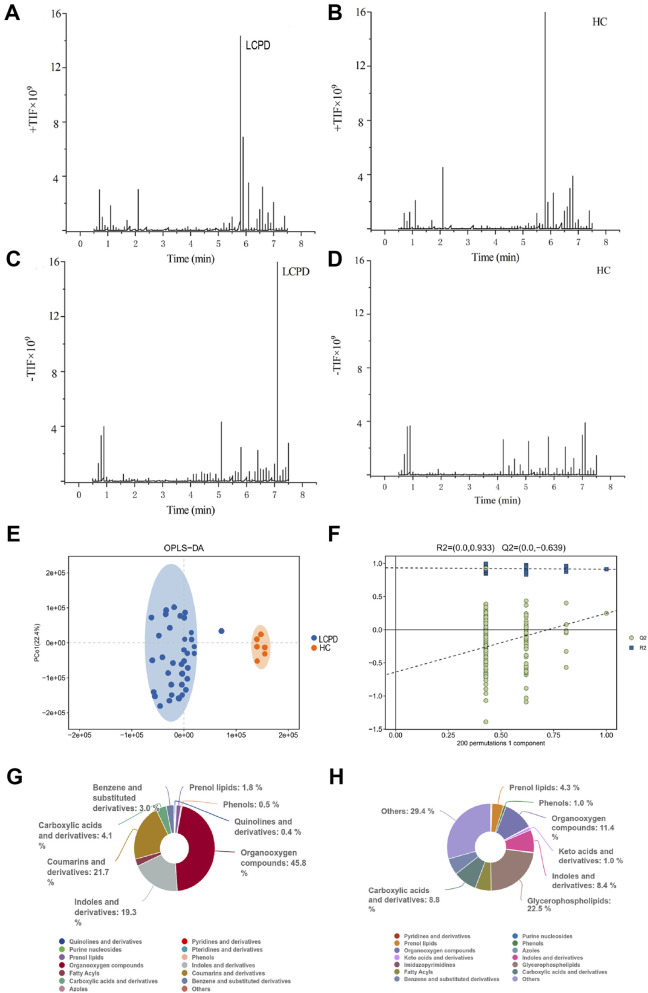
Metabolomic Profiling Reveals Distinct Metabolic Signatures in LCPD **(A,B)** Representative base peak chromatograms in positive ion mode (+TIC) for LCPD **(A)** and healthy control **(B)** samples. **(C,D)** Representative base peak chromatograms in negative ion mode (-TIC) for LCPD **(C)** and healthy control **(D)** samples. **(E)** OPLS-DA score plot showing separation between LCPD patients (n = 36) and healthy controls (n = 6). **(F)** OPLS-DA model validation by permutation test (200 permutations). **(G,H)** Pie charts showing the distribution of metabolite classes in LCPD **(G)** and healthy control **(H)** samples based on the HMDB database annotation.

Comprehensive liquid chromatography-mass spectrometry (LC-MS) analysis revealed distinctive metabolic profiles between patients with Legg-Calvé-Perthes Disease (LCPD) and healthy controls (HC). Base peak chromatograms in both positive (+TIC) and negative (-TIC) ionization modes demonstrated marked differences in metabolite distribution and abundance. In positive ionization mode, LCPD samples (Figure 1A) exhibited distinctly different peak patterns compared to HC samples (Figure 1B), particularly in regions corresponding to inflammation-associated metabolites. Similar differential patterns were observed in negative ionization mode (Figures 1C,D), albeit with comparatively lower signal intensity.

To assess the discriminatory power of the identified metabolites, orthogonal partial least squares discriminant analysis (OPLS-DA) was applied to a dataset of 1,236 metabolites from 36 LCPD patients and 6 HC. The OPLS-DA model, validated by 7-fold cross-validation and 200 permutation tests, achieved robust separation between the LCPD and HC groups (Figure 1E). Model parameters showed good data fit (R2Ycum = 0.933), though the cross-validation predictive ability was low (Q2cum = −0.639), suggesting potential overfitting or limitations in distinguishing groups based purely on the overall metabolic profile with this specific model configuration. Permutation tests (200 permutations) were performed to assess the risk of overfitting (Figure 1F). Given the enhanced sensitivity of positive mode electrospray ionization (ESI) for non-acidic metabolites, data from this mode were prioritized for subsequent detailed analyses.

### 3.2 Classification and distribution of differential metabolites

Untargeted metabolomic analysis identified 1,236 metabolites (*P < 0.05, VIP > 1.0*), which were subsequently annotated using the Human Metabolome Database (HMDB). In positive ionization mode, hierarchical classification of LCPD samples revealed 528 metabolites distributed across 15 superclasses (Figure 1G). Predominant categories included organic oxidative compounds (46 metabolites), coumarins and derivatives (6 metabolites), indoles and derivatives (26 metabolites), and carboxylic acids and derivatives (89 metabolites). This distribution pattern differed notably from healthy controls (Figure 1H), where the most diverse categories were “others” (168 metabolites across 38 classes), glycerophospholipids (50 metabolites), and organic oxidative compounds (57 metabolites). The distinct metabolic signatures and clear separation between LCPD and control samples suggested fundamental alterations in metabolic processes associated with LCPD pathogenesis. Notably, an enrichment of inflammation-related metabolites and oxidative compounds was observed, indicating potential involvement of inflammatory and oxidative stress pathways in LCPD progression.

### 3.3 Distinct metabolic patterns and characterization of significantly altered metabolites

**FIGURE 2:**
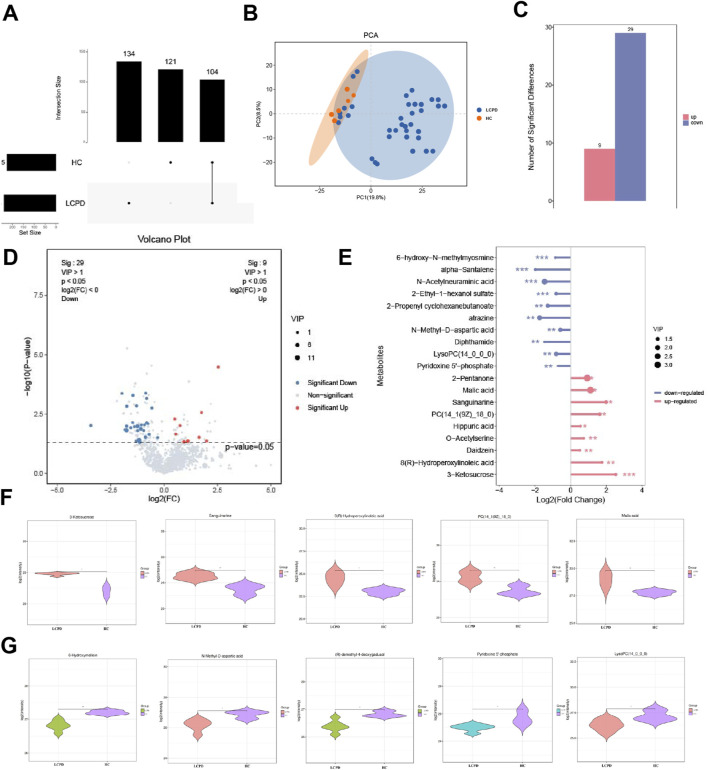
Differential Metabolite Analysis Between LCPD and Control Groups **(A)** Upset plot showing the distribution of unique and shared metabolites between LCPD and healthy control groups. **(B)** Principal component analysis (PCA) score plot demonstrating metabolic separation between groups. **(C)** Volcano plot showing differentially expressed metabolites (red: upregulated; blue: downregulated). **(D)** Heatmap of the 38 significantly differential metabolites between groups. **(E)** Bar plot showing fold changes of the 19 characteristic differential metabolites. **(F)** Violin plots showing the distribution of five key upregulated metabolites. **(G)** Box plots showing significant decreases in 6-hydroxymelatonin and N-methyl-D-aspartate levels.

Comprehensive metabolomic analysis revealed distinct metabolic patterns between LCPD patients and HC. Upset plot analysis, visualizing intersecting sets, demonstrated unique metabolic signatures in both groups (Figure 2A). Specifically, 134 unique differential metabolites were identified in the HC group and 121 in the LCPD group, with 104 metabolites shared between them. Principal component analysis (PCA) further confirmed this metabolic distinction, showing clear separation between LCPD and HC groups (Figure 2B), suggesting fundamental differences in disease-related metabolic processes.

### 3.4 Characterization of significantly altered metabolites

Based on stringent filtering criteria (*VIP ≥ 1* from OPLS-DA, and *fold change ≥ 2 or ≤ 0.5*), 38 significantly differential metabolites were identified between LCPD and HC groups (details in Supplementary Table S2). Among these, 9 metabolites were significantly upregulated, and 29 were significantly downregulated in LCPD patients (Figures 2C,D). A detailed analysis highlighted 19 of these as characteristic differential metabolites. The upregulated metabolites included 2-pentanone, malic acid, sanguinarine, PC(14:1 (9Z)/18:0), hippuric acid, O-acetylserine, genistein, 8(R)-hydroxylinoleic acid, and 3-ketoglucose. Conversely, significant downregulation was observed for 6-hydroxy-h-methylguanine, α-santalene, N-acetylneuraminic acid, 2-ethyl-1-hexyl sulfate, 2-propenylcyclohexyl butyrate, atrazine, N-methyl-D-aspartate, dipeptidamide, lysophosphatidylcholine (14:0/0:0), and pyridoxal 5′-phosphate (Figure 2E).

### 3.5 Detailed analysis of key metabolic changes

Further examination using violin plots revealed significant alterations in five key upregulated metabolites potentially involved in inflammation, vascular function, and bone health: 3-ketoglucose, sanguinarine, 8(R)-hydroxylinoleic acid, PC(14:1 (9Z)/18:0), and malic acid (Figure 2F). These compounds are known to play roles in modulating inflammatory responses, oxidative stress, and protease activity, potentially contributing to a balanced inflammatory environment and reducing inflammation-induced tissue damage. Additionally, analysis of downregulated metabolites showed significant decreases (*P < 0.01*) in 6-hydroxymelin and N-methyl-D-aspartate levels in the LCPD group (Figure 2G), suggesting altered inflammatory pathways in LCPD pathogenesis.

### 3.6 Metabolic pathway analysis reveals complex network perturbations in LCPD

**FIGURE 3:**
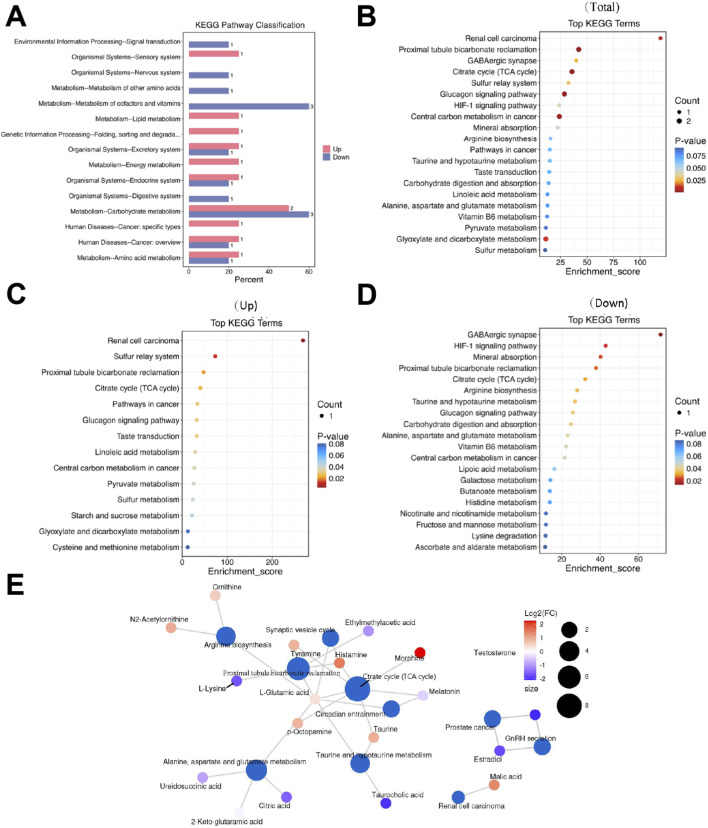
Metabolic Pathway Analysis **(A)** KEGG secondary metabolic pathway analysis showing differential pathway enrichment. **(B)** Bubble plot of the top 20 enriched pathways based on P-values. **(C)** Network analysis of upregulated pathways. **(D)** Heatmap showing suppressed metabolic pathways. **(E)** Pathway interconnection analysis showing relationships between key metabolic pathways.

Systematic classification and annotation of identified metabolites via the KEGG database indicated significant pathway alterations in LCPD. Analysis of KEGG secondary metabolic pathways demonstrated distinct patterns of metabolic dysregulation between LCPD patients and healthy controls (Figure 3A). Pathway enrichment analysis identified 65 significantly altered pathways (*P < 0.01*, *FDR < 0.05*). The top 20 most significantly altered pathways were visualized in a bubble plot based on P-values (Figure 3B). KEGG analysis revealed strong associations with multiple pathways during LCPD progression, including renal cell carcinoma signaling, sulfur transfer systems, proximal tubule bicarbonate reabsorption, and the citric acid (TCA) cycle (Figure 3C). Several pathways exhibited significant suppression, notably the GABAergic synapse, HIF-1 signaling pathway, mineral absorption, proximal tubule bicarbonate recovery, TCA cycle, and arginine biosynthesis (Figure 3D). Network analysis illustrated extensive interconnections between metabolites and multiple pathways (Figure 3E). The TCA cycle emerged as a central hub in cellular energy metabolism, with strong connections to oxidative stress and energy production through metabolites such as citrate and malate. Tyrosine metabolism pathway analysis highlighted the production of key neurotransmitters and inflammatory mediators, including tyramine and histamine, which may contribute to the inflammatory response in avascular necrosis of the femoral head.

### 3.7 Protective effects of 3-ketoglucose and sanguinarine on LPS-Induced HUVEC dysfunction

The protective effects of 3-ketoglucose and sanguinarine against LPS-induced HUVEC injury were investigated.

**FIGURE 4:**
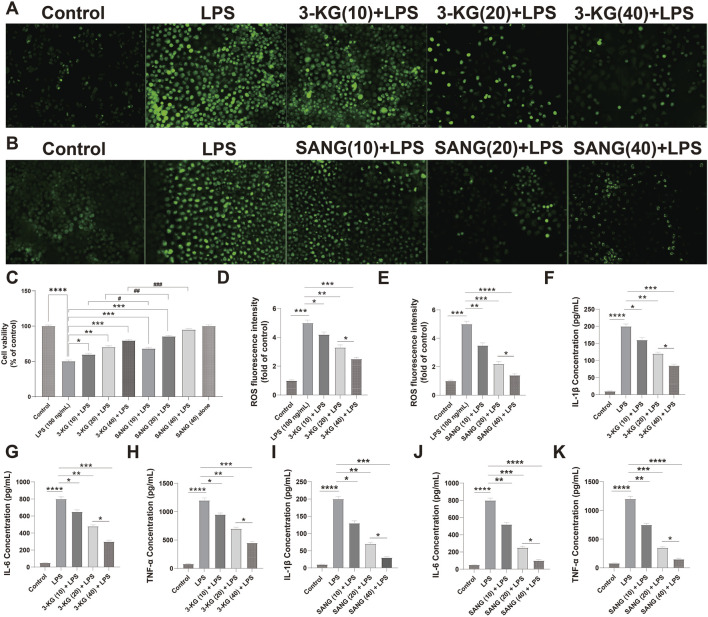
Protective and dose-dependent effects of 3-ketoglucose and sanguinarine on LPS-induced HUVEC dysfunction **(A,B)** Representative fluorescence images showing intracellular ROS levels. **(C)** Cell viability assessed by CCK-8 assay. **(D,E)** Quantification of ROS fluorescence intensity after treatment with 3-ketoglucose **(D)** and sanguinarine **(E)**. **(F–H)** ELISA analysis of IL-1β (F), IL-6 **(G)**, and TNF-α **(H)** levels after treatment with different concentrations of 3-ketoglucose. **(I–K)** ELISA analysis of IL-1β **(I)**, IL-6 **(J)**, and TNF-α **(K)** levels after treatment with different concentrations of sanguinarine. Data are presented as mean ± SD from three independent experiments. Statistical significance is indicated as *P < 0.05, **P < 0.01, ***P < 0.001, ****P < 0.0001.

Next, we evaluated their protective effects against LPS-induced injury. As expected, LPS treatment (100 ng/mL) significantly reduced HUVEC viability (P < 0.0001). Pre-treatment with either 3-ketoglucose or sanguinarine mitigated this damage in a clear dose-dependent manner. While the 10 μg/mL dose provided moderate protection, the 20 μg/mL and 40 μg/mL doses resulted in a significant recovery of cell viability, with the highest dose (40 μg/mL) being most effective. Notably, at each corresponding concentration, sanguinarine exhibited significantly stronger cytoprotective effects than 3-ketoglucose (P < 0.05) (Figure 4C).

A similar dose-dependent pattern was observed in the assessment of oxidative stress (Figures 4A,B,D–E). LPS stimulation led to a dramatic increase in intracellular ROS levels (P < 0.0001). Pre-treatment with both compounds dose-dependently suppressed this ROS production. Sanguinarine was again more potent, with the 40 μg/mL dose almost completely normalizing ROS levels. Importantly, neither 3-KG nor SANG alone at 40 μg/mL induced any change in basal ROS levels.

Finally, the anti-inflammatory properties of the compounds were confirmed by ELISA (Figures 4F–K). LPS treatment caused a massive release of pro-inflammatory cytokines IL-1β, IL-6, and TNF-α (all P < 0.0001). Pre-treatment with 3-KG and SANG significantly and dose-dependently attenuated the secretion of all three cytokines. The inhibitory effect was most pronounced at the 40 μg/mL dose, with sanguinarine once again demonstrating superior anti-inflammatory activity compared to 3-ketoglucose.

### 3.8 Transcriptomic profiling reveals distinct gene expression patterns in treated HUVECs

**FIGURE 5:**
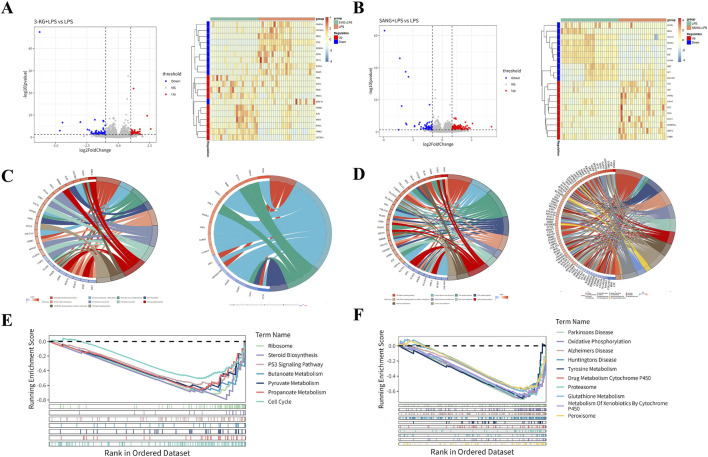
Transcriptomic Analysis of HUVECs **(A)** Volcano plots and heatmaps showing differential gene expression patterns in the 3-KG + LPS versus LPS and SANG + LPS versus LPS treatment groups. **(B)** GO and KEGG pathway enrichment analysis results (chord diagrams) for the 3-KG + LPS versus LPS comparison. **(C)** GO and KEGG pathway enrichment analysis results (chord diagrams) for the SANG + LPS versus LPS comparison. **(D)** GSEA enrichment plots showing key molecular signatures for both the 3-KG + LPS and SANG + LPS treatment comparisons against the LPS group.

Transcriptome sequencing analysis (Dataset: GSE244375) revealed significant alterations in gene expression profiles between HUVEC treatment groups. Differential gene expression analysis was performed with filtering criteria set to a p-value <0.05 and an absolute log2 fold change (|log2FC|) > 1. In the comparison between the 3-KG + LPS group and the LPS group, a total of 132 differentially expressed genes (DEGs) were identified, with 64 genes upregulated and 68 genes downregulated (Figure 5A). Similarly, in the comparison between the SANG + LPS group and the LPS group, 345 DEGs were identified, with 286 genes upregulated and 59 genes downregulated. These distinct sets of differential gene expressions for each respective comparison were illustrated by volcano plots (Figure 5B), where the horizontal axis represents log2FC and the vertical axis represents -log10 (p-value); dashed lines indicate the thresholds for p-value = 0.05 and |log2FC| = 1, with downregulated genes shown on the left and upregulated genes on the right for each comparison. Heatmaps were generated to visualize the top 10 upregulated and downregulated genes from these analyses, showing gene clustering (left side of heatmap) and expression changes, where colors closer to red indicate more significant upregulation and colors closer to blue indicate more significant downregulation (Figures 5A,B). Among the differentially expressed genes identified through this transcriptomic analysis, *NFKB1* (encoding *NF-κB* p65), *VCAM1*, and *NOS3* (encoding *eNOS*) were identified as showing differential expression, aligning with their roles in inflammatory and vascular pathways, and these were selected for further detailed validation as described later.

To further elucidate the biological functions and pathways affected by 3-KG and SANG treatments in the context of LPS stimulation, Gene Ontology (GO) enrichment analysis, Kyoto Encyclopedia of Genes and Genomes (KEGG) pathway enrichment analysis, and Gene Set Enrichment Analysis (GSEA) were performed.

For HUVECs pre-treated with 3-ketoglucose (the 3-KG + LPS group), Gene Ontology (GO) analysis of the differentially expressed genes identified enrichment primarily in four biological process (BP) terms. Notable among these were “Negative Regulation Of Monocyte Chemotaxis” and pathways related to pain perception, such as “Detection Of Stimulus Involved In Sensory Perception Of Pain.” These findings are visualized in a chord diagram (Figure 5C). For the SANG + LPS group, a total of 26 pathways were enriched, comprising 5 BP terms, 16 cellular component (CC) terms, and 5 molecular function (MF) terms. The top 5 enriched BP terms included Ossification, DNA Recombinase Assembly, DNA Repair Complex Assembly, Axon Guidance, and Neuron Projection Guidance. Among the top 5 CC terms were Growth Cone, Site Of Polarized Growth, Dendritic Spine, Neuron Spine, and Endoplasmic Reticulum Lumen. The top 5 MF terms included Monoatomic Ion Gated Channel Activity, Small Molecule Sensor Activity, Calmodulin Binding, DNA Strand Exchange Activity, and ATP-Dependent DNA Damage Sensor Activity. These findings are represented in a chord diagram (Figure 5D).

KEGG pathway analysis for the 3-KG + LPS group identified 14 enriched pathways, with the top 10 pathways including Chemical carcinogenesis - DNA adducts, Folate biosynthesis, and Cholesterol metabolism (Figure 5C). In the SANG + LPS group, 31 KEGG pathways were enriched. The top 10 significantly enriched pathways included NF-kappa B signaling pathway, Protein digestion and absorption, Ras signaling pathway, and alpha-Linolenic acid metabolism, among others (Figure 5D).

GSEA was performed using a threshold of ||NES|>1 and P-value <0.05 to identify significantly enriched pathways. For the 3-KG + LPS group, 7 pathways were enriched, and all are presented. These included Ribosome, Steroid Biosynthesis, P53 Signaling Pathway, Butanoate Metabolism, Pyruvate Metabolism, Propanoate Metabolism, and Cell Cycle (Figure 5E). In the SANG + LPS group, the top 10 significantly enriched pathways are visualized. These included Parkinson’s Disease, Oxidative Phosphorylation, Alzheimer’s Disease, Huntington’s Disease, Tyrosine Metabolism, Drug Metabolism Cytochrome P450, Proteasome, Glutathione Metabolism, Metabolism Of Xenobiotics By Cytochrome P450, and Peroxisome (Figure 5F).

### 3.9 Molecular mechanisms of 3-ketoglucose and sanguinarine in LPS-Induced endothelial inflammation

**FIGURE 6:**
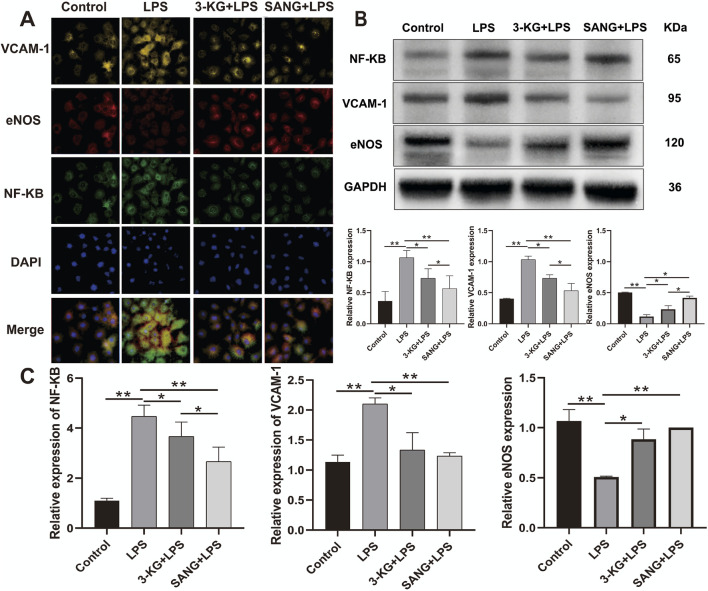
Molecular Mechanism Analysis **(A)** Representative immunofluorescence images showing expression of *NF-κB*, *eNOS,* and *VCAM-1*. Scale bar = 50 μm. **(B)** Western blot banding and grayscale value statistics **(C)** RT-PCR analysis of gene expression levels. Data are presented as mean ± SD from three independent experiments. *P < 0.05, **P < 0.01.

To validate the protective effects against LPS-induced endothelial inflammation, the expression of key regulatory proteins was examined by immunofluorescence staining. Analysis revealed significant modulation of NF-κB, eNOS, and VCAM-1 expression across treatment groups. LPS stimulation markedly increased NF-κB and VCAM-1 expression while decreasing eNOS levels compared to control conditions. Treatment with either 3-ketoglucose or sanguinarine effectively reversed these changes, with sanguinarine demonstrating superior modulatory effects (Figure 6A). Western blot analysis quantitatively confirmed these observations. LPS treatment significantly elevated NF-κB and VCAM-1 protein levels (both P < 0.01) while reducing eNOS expression. Both 3-ketoglucose and sanguinarine treatment significantly attenuated these LPS-induced changes (P < 0.01). Notably, sanguinarine exhibited more potent effects than 3-ketoglucose (P < 0.05) in normalizing protein expression levels (Figure 6B). RT-PCR analysis of corresponding gene expression levels corroborated the protein-level findings. LPS treatment significantly upregulated NF-κB and VCAM-1 gene expression (both P < 0.01) while downregulating eNOS. Both 3-ketoglucose and sanguinarine treatment effectively reversed these transcriptional changes (P < 0.05), with parallel effects observed at both mRNA and protein levels (Figure 6C). The consistent findings across multiple analytical methods strengthen the evidence for the protective mechanisms of these compounds in endothelial inflammation.

### 3.10 3-Ketoglucose and sanguinarine increase serum ALP and decrease serum TG in MPS rats

**FIGURE 7:**
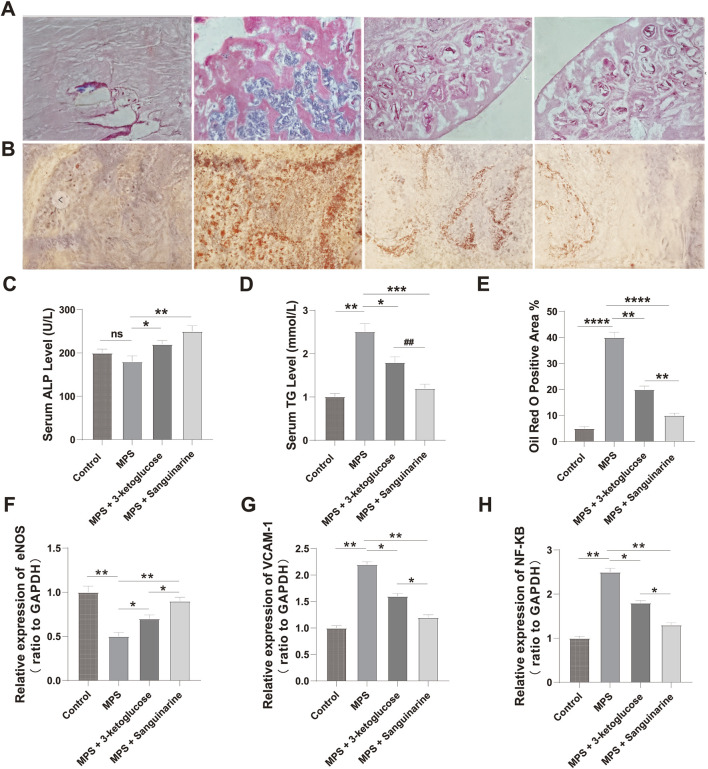
3-ketoglucose and Sanguinarine ameliorate steroid-induced osteonecrosis of the femoral head in rats **(A)** Representative images of H&E staining showing bone trabecular structure and necrosis. **(B)** Representative images of Oil Red O staining showing lipid droplet accumulation. **(C)** Quantification of serum ALP levels. **(D)** Quantification of serum TG levels. **(E)** Quantitative analysis of the Oil Red O-positive area. **(F–H)** Relative mRNA expression levels of Nos3 (eNOS) **(F)**, Vcam1 **(G)**, and Nfkb1 **(H)** in femoral head tissues. Data in bar charts are presented as mean ± SD (n = 10). ns, not significant. **P < 0.01, ****P < 0.0001 vs. the Control group. *P < 0.05, **P < 0.01, ***P < 0.001, ****P < 0.0001 vs. the MPS group. ##P < 0.01 vs. the 3-ketoglucose group.

We measured serum ALP and TG levels in rats after 12 weeks of intervention. As shown in Figure 7C, the ALP level in the MPS group was lower than that in the Control group, but the difference was not statistically significant (P > 0.05). However, we detected significantly higher ALP levels in rats treated with 3-ketoglucose and Sanguinarine compared to the MPS group (P < 0.05). As shown in Figure 7D, the serum TG level in the MPS group was significantly higher than that in the Control group (P < 0.05), while treatment with 3-ketoglucose and Sanguinarine markedly reduced its production.

### 3.11 3-Ketoglucose and sanguinarine prevent bone loss and adipogenesis in ONFH rats

We evaluated the effects of the treatments on the histomorphology of the femoral head using H&E and Oil Red O staining. The results showed significant adipose tissue invasion and obvious subchondral necrosis in the trabecular bone of the MPS group. In contrast, these pathological changes were not observed in the MPS + 3-ketoglucose and MPS + Sanguinarine groups, where only a few smaller adipocytes and empty lacunae were present (Figures 7A,B,E).

### 3.12 Treatments modulate inflammatory and endothelial pathways at the gene level

To validate our *in vitro* findings at the gene expression level, we performed qRT-PCR analysis on femoral head tissues. The results showed that, compared to the Control group, the MPS model group had significantly upregulated mRNA expression of the pro-inflammatory genes Nfkb1 and Vcam1, and significantly downregulated expression of the endothelial-protective gene Nos3 (eNOS) (Figures 7F–H). Consistent with our previous findings, treatment with both 3-ketoglucose and sanguinarine effectively reversed these MPS-induced changes. Specifically, both treatments led to a significant decrease in Nfkb1 and Vcam1 mRNA levels and a significant increase in Nos3 expression. Notably, the effect of sanguinarine was more pronounced than that of 3-ketoglucose in modulating these genes, which aligns perfectly with the trends observed at the cellular level.

## 4 Discussion

This comprehensive investigation, integrating metabolomic profiling, transcriptome analysis, and molecular validation, offers significant new insights into the pathophysiological mechanisms of Legg-Calvé-Perthes Disease (LCPD) and identifies promising avenues for therapeutic strategies. The core findings of this study center on the distinct metabolic disturbances in LCPD patients compared to healthy controls, the identification of specific metabolites with potential diagnostic and therapeutic value, and the elucidation of their protective effects on endothelial cells, a key component in LCPD pathology. These discoveries advance our understanding beyond traditional clinical and imaging-based approaches to LCPD (Laine et al., 2021; Rodríguez-Olivas et al., 2022; Ng et al., 2024).

The metabolomic analysis successfully distinguished LCPD patients from healthy controls, identifying 38 significantly different metabolites. This finding aligns with recent work demonstrating similar metabolic perturbations in bone disorders using high-resolution mass spectrometry (Petrova et al., 2021). Specifically, (Bai et al., 2025) highlighted the potential of metabolomic profiling to uncover novel biomarkers in pediatric orthopedic conditions like LCPD (Bai et al., 2025).

However, this study provides novel metabolic signatures specific to pediatric LCPD, notably the upregulation of 3-ketoglucose and sanguinarine, which, to our knowledge, have not been previously reported in this disease context. The identification of 3-ketoglucose is particularly interesting; though its direct role in LCPD is new, its reductase has been studied in *Agrobacterium tumefaciens*. Sanguinarine, a plant alkaloid, has been investigated for various biological activities, including anticancer properties (Lou et al., 2021), but its role in LCPD and endothelial protection is a novel finding of this study. These discoveries expand the current understanding of LCPD pathogenesis beyond traditional concepts. While earlier studies suggested the involvement of inflammatory pathways in LCPD progression (Leroux et al., 2018), this research bridges a critical gap by demonstrating clear links between metabolic dysregulation, specifically the upregulation of 3-ketoglucose and sanguinarine, and inflammatory processes, particularly through the modulation of *NF-κB* signaling pathways.

KEGG pathway analysis underscored the complexity of LCPD, revealing significant enrichment of multiple metabolic and inflammatory pathways, including the TCA cycle and inflammatory signaling cascades. This finding is consistent with studies highlighting the interconnection between energy metabolism and inflammation in various bone disorders (Huang et al., 2022). The implication of the renal cell carcinoma signaling pathway and sulfur transfer systems in LCPD represents a novel discovery. While the importance of sulfur metabolism in bone health has been previously noted (Ng et al., 2024), this study is the first to suggest its potential role in the progression of LCPD.

Perhaps the most significant contribution of this study is the demonstration of the protective effects of 3-ketoglucose and sanguinarine on endothelial function. Given that profound vascular dysfunction is a critical element in LCPD pathogenesis (Khayat et al., 2017), and recent evidence points to endothelial dysfunction playing a central role (Li et al., 2021),these findings are highly relevant. Liu et al. (2024) recently emphasized the interplay between endothelial dysfunction and inflammatory pathways in pediatric bone disorders, supporting our observation that 3-ketoglucose and sanguinarine mitigate LPS-induced inflammation and oxidative stress in HUVECs(Di Marcello et al., 2022). This protective mechanism appears to involve the modulation of key inflammatory and vascular pathways, including NF-κB, eNOS, and *VCAM-1*. The use of HUVECs as an *in vitro* model is well-established for studying endothelial cell biology and dysfunction in various pathological contexts, including inflammation and angiogenesis (Elomaa et al., 2022; Yang et al., 2023). The observed reduction in ROS production and pro-inflammatory cytokines (IL-1β, IL-6, TNF-α) further supports the therapeutic potential of these metabolites.

Crucially, our study extends these significant *in vitro* observations to an *in vivo* setting, providing a powerful validation of their therapeutic potential. Using a well-established rat model of steroid-induced osteonecrosis, which shares key pathological features with LCPD such as vascular disruption and inflammation, we confirmed that both 3-ketoglucose and sanguinarine offer significant protection. The *in vivo* results provided strong corroboration for our cellular findings: treatment not only ameliorated bone loss and adipogenesis in the femoral head (Figures 7A,B,E) but also recapitulated the molecular changes observed in HUVECs. The downregulation of pro-inflammatory genes Nfkb1 and Vcam1 and the upregulation of the endothelial-protective gene Nos3 (eNOS) in the femoral head tissues of treated rats (Figures 7F–H) directly mirrors the molecular modulation observed in LPS-stimulated HUVECs (Figure 6C). This consistency between the *in vitro* and *in vivo* data provides a much stronger foundation for the therapeutic potential of these metabolites, bridging the gap from a cellular mechanism to a potential whole-organism effect.

The transcriptomic analysis provided a broader view of the molecular changes, complementing other molecular studies and highlighting the multi-faceted nature of LCPD pathogenesis. Our findings on altered metabolites that protect endothelial cells align with research identifying different circulating biomarkers, such as endothelial microparticles (EMPs), which similarly point to severe endothelial injury in LCPD (Li et al., 2021).

Furthermore, our work complements studies on other molecular regulators, such as microRNAs. For instance, molecules like miR-150 are known to regulate bone cell differentiation (Moussa et al., 2021), a critical process for healing, while other research has identified broader miRNA signatures in the plasma of LCPD patients (Huang et al., 2022). Collectively, these different lines of evidence—from metabolomics, proteomics, and transcriptomics—are building a more comprehensive molecular picture of LCPD, paving the way for integrated diagnostic and therapeutic strategies.

Despite the significant findings, this study has limitations that warrant consideration. Firstly, a significant limitation of this study is the small sample size of the healthy control group (n = 6) relative to the LCPD group (n = 36). Although age- and sex-matching was performed and a power analysis (G*Power 3.1; effect size = 0.8, α = 0.05, power = 0.9) suggested the sample size was adequate for detecting large effect sizes, this limited number of controls may reduce the statistical power for detecting more subtle metabolic changes and could affect the robustness and generalizability of the OPLS-DA model and some metabolomic findings. Therefore, the metabolic signatures identified, particularly those reliant on comparisons with this small control cohort, warrant cautious interpretation and require validation in future studies with larger, well-balanced cohorts to confirm their broader applicability.

Secondly, it is important to note that while the OPLS-DA model demonstrated a good fit to the current dataset (R2Ycum = 0.933), the negative Q2cum value (−0.639) indicates limited predictive capability for group separation in cross-validation with this model configuration. This could be attributed to factors such as the inherent complexity of LCPD metabolic profiles, the relatively small size of the control group, or the specific parameters chosen for the model. Nevertheless, the identification of individual differential metabolites was further supported by their VIP scores and univariate statistical significance, and key findings were subsequently validated through *in vitro* experiments. Furthermore, it is important to acknowledge that the steroid-induced ONFH model, while widely used, may not fully replicate the idiopathic and developmental nature of LCPD in children.

Furthermore, the cross-sectional design precludes the analysis of temporal changes in metabolic profiles throughout disease stages (necrosis, fragmentation, reossification, and remodeling) or in response to treatment, a limitation also acknowledged in the context of longitudinal investigations. While key metabolic pathways were identified, further research is needed to fully elucidate the intricate molecular mechanisms linking these metabolic alterations to LCPD progression, as suggested by the need for mechanistic studies.

The cross-sectional design precludes the analysis of temporal changes in metabolic profiles throughout disease stages (necrosis, fragmentation, reossification, and remodeling (Ng et al., 2024) or in response to treatment, a limitation also acknowledged in the context of longitudinal investigations (Leibold et al., 2023; Schwert, 2023). While key metabolic pathways were identified, further research is needed to fully elucidate the intricate molecular mechanisms linking these metabolic alterations to LCPD progression, as suggested by the need for mechanistic studies (Selberg et al., 2021).

Several future research directions emerge from this work. Longitudinal studies are crucial to track metabolic changes across different stages of LCPD (early to healing) and in response to interventions, providing valuable temporal insights, a need suggested by other research focusing on the disease course. Advanced techniques like single-cell sequencing and spatial metabolomics could offer a more granular understanding of cellular heterogeneity and the metabolic landscape within the affected femoral head, approaches whose value is increasingly recognized. Crucially, clinical validation studies are necessary to assess the safety and efficacy of 3-ketoglucose and sanguinarine as potential therapeutic agents in LCPD patients, adhering to rigorous clinical trial protocols similar to those established in therapeutic research. Furthermore, validating the identified metabolic signatures (e.g., 3-ketoglucose, sanguinarine, 6-hydroxymelin, N-methyl-D-aspartate) as diagnostic and prognostic biomarkers in larger, multicenter patient cohorts is essential. Such biomarker validation studies have proven valuable in other pediatric orthopedic conditions (Rodríguez-Olivas et al., 2022).

In conclusion, this study provides novel and comprehensive insights into the metabolic and molecular underpinnings of LCPD. The identification of distinct metabolic signatures in peripheral blood, coupled with the demonstrated therapeutic potential of 3-ketoglucose and sanguinarine in mitigating endothelial dysfunction and inflammation, represents a significant advancement in understanding this enigmatic pediatric hip disorder. These findings not only enhance our comprehension of the complex interplay between metabolic dysregulation, inflammation, and vascular compromise in LCPD but also lay a strong foundation for the development of innovative diagnostic tools and more effective therapeutic interventions. While further validation and research are imperative, this work offers hope for improving outcomes for children affected by LCPD.

## 5 Conclusion

Through integrated metabolomic and transcriptomic analyses, this study reveals the unique metabolic signature of LCPD and provides the first evidence that 3-ketoglucose and sanguinarine exert protective effects by mitigating endothelial dysfunction and inflammation. These findings offer a new molecular perspective for understanding LCPD’s pathogenesis and lay a solid foundation for developing novel diagnostic biomarkers and therapeutic targets for the disease.

## Data Availability

The original contributions presented in the study are publicly available. This data can be found here: https://www.ncbi.nlm.nih.gov/bioproject/PRJNA1333660/.
