# Supplementary material for: Metabolomic profiling reveals novel biomarkers and therapeutic targets in Legg-Calvé-Perthes disease: a comprehensive analysis of peripheral blood and endothelial function
Source: Front Physiol. 2025 Oct 1;16:1641445. doi: 10.3389/fphys.2025.1641445 (PMC12521442; doi:10.3389/fphys.2025.1641445)
Supplement: Supplementary file 1 [file Table1.docx]

**Table S1. Primers used in realtime PCR （cell）**

| **Gene** | **Forward Primer (5' to 3')** | **Reverse Primer (5' to 3')** |
| --- | --- | --- |
| NF-κB | GCAGCACTACTTCTTGACCACC | TCTGCTCCTGAGCATTGACGTC |
| VCAM-1 | GCTATGAGGATGGAAGACTCTGG | ACTTGTGCAGCCACCTGAGATC |
| eNOS | TGGAGGAGCACGAGTGAACG | TGGTGGTGAAGGAGGAGGTC |
| GAPDH | CATGGGTGTCAACGGATTTGGC | GAGTGGGAGTATGTCGTCTC |
