# Supplementary material for: Metabolomic profiling reveals novel biomarkers and therapeutic targets in Legg-Calvé-Perthes disease: a comprehensive analysis of peripheral blood and endothelial function
Source: Front Physiol. 2025 Oct 1;16:1641445. doi: 10.3389/fphys.2025.1641445 (PMC12521442; doi:10.3389/fphys.2025.1641445)
Supplement: Supplementary file 2 [file Table2.docx]

**Table S2. Primer sequences used for qRT-PCR in rats.**

| Gene (Rat) | Forward Primer (5' to 3') | Reverse Primer (5' to 3') |
| --- | --- | --- |
| Nfkb1 | AGCCAGAGGTTATTCAGCCCT | GCAACTGGAAAGAGGCTGTC |
| Vcam1 | TGACTGCAGAACCCGATTTC | AGCACCGTCAACATTGAACC |
| Nos3 | CGGCTACCAGCAGGAATCAG | TCCAGGAGGGTCTCGTACTG |
| Gapdh | GGCACAGTCAAGGCTGAGAATG | ATGGTGGTGAAGACGCCAGTA |
